# Perinatal Exposure to Traffic-Related Air Pollution and Atopy at 1 Year of Age in a Multi-Center Canadian Birth Cohort Study

**DOI:** 10.1289/ehp.1408700

**Published:** 2015-03-31

**Authors:** Hind Sbihi, Ryan W. Allen, Allan Becker, Jeffrey R. Brook, Piush Mandhane, James A. Scott, Malcolm R. Sears, Padmaja Subbarao, Tim K. Takaro, Stuart E. Turvey, Michael Brauer

**Affiliations:** 1 School of Population and Public Health, University of British Columbia, Vancouver, British Columbia, Canada; 2 Faculty of Health Sciences, Simon Fraser University, Burnaby, British Columbia, Canada; 3 Department of Immunology, Faculty of Medicine, University of Manitoba, Winnipeg, Manitoba, Canada; 4 Air Quality Research Division, Environment Canada, Toronto, Ontario, Canada; 5 Department of Pediatrics, Faculty of Medicine and Dentistry, University of Alberta, Edmonton, Alberta, Canada; 6 Dalla Lana School of Public Health, University of Toronto, Toronto, Ontario, Canada; 7 Department of Medicine, Faculty of Health Sciences, McMaster University, Hamilton, Ontario, Canada; 8 Hospital for Sick Children, Department of Paediatrics, Faculty of Medicine, University of Toronto, Toronto, Ontario, Canada; 9 BC Children’s Hospital and Child and Family Research Institute, Department of Paediatrics, Faculty of Medicine, University of British Columbia, Vancouver, British Columbia, Canada

## Abstract

**Background:**

The role of traffic-related air pollution (TRAP) exposure in the development of allergic sensitization in children is unclear, and few birth cohort studies have incorporated spatiotemporal exposure assessment.

**Objectives:**

We aimed to examine the association between TRAP and atopy in 1-year-old children from an ongoing national birth cohort study in four Canadian cities.

**Methods:**

We identified 2,477 children of approximately 1 year of age with assessment of atopy for inhalant (*Alternaria*, *Der p*, *Der f*, cat, dog, cockroach) and food-related (milk, eggs, peanuts, soy) allergens. Exposure to nitrogen dioxide (NO_2_) was estimated from city-specific land use regression models accounting for residential mobility and temporal variability in ambient concentrations. We used mixed models to examine associations between atopy and exposure during pregnancy and the first year of life, including adjustment for covariates (maternal atopy, socioeconomic status, pets, mold, nutrition). We also conducted analyses stratified by time-location patterns, daycare attendance, and modeled home ventilation.

**Results:**

Following spatiotemporal adjustment, TRAP exposure after birth increased the risk for development of atopy to any allergens [adjusted odds ratio (aOR) per 10 μg/m^3^ NO_2_ = 1.16; 95% CI: 1.00, 1.41], but not during pregnancy (aOR = 1.02; 95% CI: 0.86, 1.22). This association was stronger among children not attending daycare (aOR = 1.61; 95% CI: 1.28, 2.01) compared with daycare attendees (aOR = 1.05; 95% CI: 0.81, 1.28). Trends to increased risk were also found for food (aOR = 1.17; 95% CI: 0.95, 1.47) and inhalant allergens (aOR = 1.28; 95% CI: 0.93, 1.76).

**Conclusion:**

Using refined exposure estimates that incorporated temporal variability and residential mobility, we found that traffic-related air pollution during the first year of life was associated with atopy.

**Citation:**

Sbihi H, Allen RW, Becker A, Brook JR, Mandhane P, Scott JA, Sears MR, Subbarao P, Takaro TK, Turvey SE, Brauer M. 2015. Perinatal exposure to traffic-related air pollution and atopy at 1 year of age in a multi-center Canadian birth cohort study. Environ Health Perspect 123:902–908; http://dx.doi.org/10.1289/ehp.1408700

## Introduction

The incidence of allergic diseases has increased sharply, especially for people living in urban areas, which raises an important public health concern given the trend to urbanization worldwide (D’Amato et al. 2010). Although associations between exposure to air pollution derived from traffic emissions and allergic exacerbations have been demonstrated, the potential role of traffic-related air pollution (TRAP) in the onset of allergic diseases is uncertain (Heinrich and Wichmann 2004).

Several epidemiological studies have reported associations between atopy or other allergic phenotypes and exposure to some TRAP exposure surrogates, including nitrogen dioxide (NO_2_) (Brauer et al. 2007; Gruzieva et al. 2012; Janssen et al. 2003; Morgenstern et al. 2008). However, a number of other studies have not observed these positive associations (Gehring et al. 2010; Oftedal et al. 2007). Differences in TRAP exposure assessment approaches are one possible explanation for these divergent findings.

Despite the importance of early-life exposures in the development of allergy, information on the effect of air pollution exposure, particularly during pregnancy, on allergic responses early in life has rarely been assessed (Aguilera et al. 2013; Esplugues et al. 2011; Mortimer et al. 2008), and birth cohort studies addressing this relationship are rare (Bråbäck and Forsberg 2009). Exposure assessment methods have evolved from self-reported measures (e.g., proximity) (Janssen et al. 2003) to atmospheric dispersion (Oftedal et al. 2007) and land use regression (LUR) models capturing within-city air pollution variations (Brauer et al. 2007; Gehring et al. 2010). Most LUR models consider annual average concentrations, which may over- or underestimate personal exposures because traffic markers such as NO_2_, are highly variable in time and space (Mölter et al. 2010). Recently the temporal specificity of LUR models has been improved by applying forward (Morgenstern et al. 2008) or backward (Brauer et al. 2007; Gehring et al. 2010) time trends from fixed-site monitoring stations to the LUR predictions. However, given the potential importance of specific short-duration periods of development, refined approaches to capture fine-scale seasonal variability are needed (Mortimer et al. 2008). Exposure assessment is further complicated by the mobility of study participants, a consideration seldom addressed and rarely measured in large cohort studies (Arrandale et al. 2011), yet one with considerable influence on personal exposures (Nethery et al. 2009). The inclusion of exposure in other locations where children spend a significant amount of time, such as daycare facilities, is likely to increase the precision of the exposure estimates. Finally, all prior studies have used ambient concentrations at a given location to estimate exposure, although most time is usually spent indoors, especially in the early years of life (Leech et al. 2002). Considering that indoor infiltration for some pollutants can vary significantly between residences (Clark et al. 2010), substantial exposure misclassification may be present in prior studies.

To address these limitations we examined the association between exposure to TRAP and the development of atopy in a population-based multi-center birth cohort, the Canadian Healthy Infant Longitudinal Development (CHILD) study. We estimated exposure to NO_2_ as a surrogate for TRAP during pregnancy and the first year of life while accounting for both spatial and temporal variability in ambient concentrations. In stratified analyses we evaluated the influence of time–location patterns, daycare attendance, and modeled estimates of infiltration.

## Methods

*Study population*. The CHILD study (http://www.canadianchildstudy.ca) is a prospective longitudinal national birth cohort that recruited over 3,600 families between 2008 and 2012 in four Canadian cities [Vancouver (2.31 million inhabitants), Edmonton (1.16 million), Winnipeg (0.73 million), and Toronto (5.58 million)] (Takaro et al. 2015). Participants had to be pregnant, ≥ 18 years of age (19 years in Vancouver), reside in reasonable proximity to a recruitment center (originally set about 50 km distance from the study center), communicate in English, provide informed consent, intend to give birth at one of the recruitment centers, and able to provide valid personal contact information as well as two alternate contact individuals. Eligible infants had to be born at ≥ 35.5 weeks gestation with weight ≥ 2,500 g. Infants were excluded under any of the following criteria: conception via *in vitro* fertilization, product of a multiple-gestation pregnancy, major congenital abnormalities, spending < 80% of nights in the index home. Recruitment ideally occurred at or soon after the routine 18-week ultrasound examination, but a significant proportion was recruited in later pregnancy (34% between 24 and 30 weeks, and 31% after 30 weeks of gestation). Each study center (universities and hospitals) obtained ethics approval from their governing health and ethics board, and the CHILD study was reviewed and approved by the Hamilton Integrated Ethics Board (certificate number 07-2929).

The start of follow-up was defined as the date of conception based on the expected and actual dates of delivery. End of follow-up was defined as the earliest of either 15 October 2013 or when the participating child was assessed with skin allergy testing at approximately 1 year of age.

*Skin-prick tests*. To determine individual allergen sensitization, epicutaneous skin tests were administered to each infant at approximately 1 year of age for the six inhalant [*Alternaria alternata*, cat hair, dog epithelium, house dust mites (*Dermatophagoides pteronyssinus* and *D. farinae*), German cockroach] and four food (whole cow’s milk, egg white, soybean, peanut) allergens. Histamine 1 mg/mL and glycerin were used as positive and negative controls. To define maternal atopy, each mother was tested with a panel of allergens: *Alternaria alternata*, cat hair, dog epithelium, house dust mites, German cockroach, *Cladosporium* sp., *Penicillium* mixed, *Aspergillus fumigatus*, Midwest trees, grass mix, weeds, mixed ragweed, and peanut. Atopic status was determined using a positive response to any allergen. We defined two clusters of atopic responses in the children—inhalant allergy and food allergy.

Participants reporting use of antihistamines during the 7 days before the date of skin-prick test were excluded. The wheal responses were measured at 10 (histamine) and 15 (allergen) min. We averaged the maximum diameter and its orthogonal, and defined a positive response as a wheal diameter ≥ 2 mm greater than the response to the negative control. We included all participants with a positive response to histamine and no response to glycerin, or those with one or more positive responses (≥ 2 mm) to any allergen, even if there was a weak or no response to histamine. Participants with a positive response to one or more allergens but also a response to the negative control were included with adjustment for the negative control (subtraction of the mean wheal diameter of the negative control from each positive test wheal diameter). In some cases specific tests were omitted (e.g., some families declined infant peanut testing), and these specific allergens responses were recorded as missing, but all other data from that participant were included. Participants with no response to histamine and no response to any allergen were excluded, as were participants with dermatographism, when the response to the negative control was as large as any other response.

*Air pollution exposure assessment*. City-specific LUR models were developed to estimate NO_2_ concentrations and used to assign exposures at the residential locations of all participants. The models are described in detail elsewhere and were developed at different time points between 2003 and 2008 (Allen et al. 2011; Henderson et al. 2007; Jerrett et al. 2007). Models were developed using road and land use data provided by Desktop Mapping Technologies Inc. (DMTI; http://www.dmtispatial.com), as well as additional city-specific data sources (e.g., traffic counts from the city of Toronto).

Although individual models differed between locations, the variables used for model building had relatively similar grouping categories (see Supplemental Material, Table S1): *a*) land use, *b*) road and traffic, *c*) population, *d*) physical geography, and *e*) meteorology. All statistical models were consistently developed using the methodology adopted in the initial Vancouver model (Henderson et al. 2007). These models allow cost-effective, relatively precise individual predictions of TRAP at the participants’ residence.

Individual estimates of exposure were extracted for each georeferenced residence reported by participants from address at conception to the address where the participating child resided at age 1 year. Exposure to NO_2_ was assessed in two case scenarios. In the first scenario, we used the traditional approach of using only the address at the time of birth. Second, for those reporting multiple addresses we considered their exposure at every address during the study period and computed their time-weighted average exposure. All exposures were subsequently temporally adjusted based on local fixed-site ambient monitoring data on a bi-weekly basis (see Equation 1 for one 2-week interval) over the entire pregnancy and first year of life by multiplying the LUR estimate by the ratio of the contemporaneous average concentrations measured at all fixed-site governmental monitoring stations (Vancouver: *n* = 12, Toronto: *n* = 7, Edmonton: *n* = 4, and Winnipeg *n* = 2) to the annual average at the same stations for the year the model was developed:

adj. NO_2_ = NO_2(LUR)_[2-week NO_2(monitor)_/ yearly NO_2(monitor)_], [1]

where adj. NO_2_ refers to temporally adjusted individual estimate of NO_2_.

Because the LUR models did not in all cases cover the full areas where participants resided, except for Vancouver, it was necessary to impute exposures for those outside of the LUR model domain. We restricted the locations where exposures were imputed to those residences within 50 km from the municipal center, defined as the location of the city hall. Participants with home addresses located outside of a 50-km buffer from the city hall were not assigned TRAP exposure. To reduce discontinuities in exposure estimates on the periphery of the study area, we first defined core city limits within the original LUR surface where the models were applied directly to estimate exposures. For all homes outside these core city limits and within the 50-km buffer, the minimum value of the LUR surface in the core city area was assigned to homes within 100 m of highway [defined by standard road classification categories (DMTI Spatial Inc., Markham, Ontario, Canada), with categories 1 (expressway), 2 (principal highway), and 3 (secondary highway)], and the minimum value of the original LUR surface was assigned to homes located farther than 100 m away from a highway. Finally, participants with a TRAP concentration estimated for at least 75% of the time window of interest (i.e., full pregnancy or first year of life) were assigned the mean exposure of the corresponding time window.

*Time–activity patterns and daycare attendance*. The CHILD home environmental questionnaires were used to gather information on the microenvironments within and outside the home of each participating child since birth. From the 3-, 6-, and 12-month home questionnaires, we derived *a*) the average time spent away from home (i.e., time-weighted average of hours per day spent outside the house based on typical weekday and weekend time–activity patterns) that was divided into two strata by the city-specific median time away from the house, and *b*) whether the participating child ever attended a daycare or other indoor location for at least 7 hr/week or 1 hr/day regularly at any time point during the first year of life. Using these estimates we evaluated the association between TRAP exposure and atopy in stratified analyses comparing children ever/never attending daycare and children spending more time or less time than the city-specific median outside the home.

*Infiltration*. To assess the potential impact of ventilation, we adapted a model previously developed (Allen et al. 2012) to predict fine particle (particulate matter ≤ 2.5 μm; PM_2.5_) infiltration in six U.S. cities as part of the MESA Air (Multi-Ethnic Study of Atherosclerosis and Air Pollution)study. The model included information on residence characteristics and behaviors related to infiltration, including presence/use of air conditioning (AC), outdoor air temperature, window opening, and use/type of heating. As the CHILD and MESA Air questionnaires differed, we mapped the MESA Air variables onto those collected in CHILD to predict infiltration (see Supplemental Material, Table S2). For behaviors that varied seasonally, participants were asked about typical behavior in mid-summer and mid-winter. Season was defined by the Environment Canada daily temperature data (http://climate.weather.gc.ca/), collected from the same monitors from 2008 to 2013, where the warm season was defined as every 2-week period with a median temperature > 18°C, similar to the MESA Air study. We predicted home-specific seasonal infiltration efficiencies and classified homes based on the city-specific 80th percentile of infiltration efficiency for each season, where participating houses above this threshold were classified as “leaky.” As our exposure of interest was NO_2_, we used modeled particle infiltration as a surrogate for home ventilation and infiltration of TRAP in general, and assessed effect modification by infiltration in stratified analyses.

*Questionnaires and home inspections*. Covariates relating to *a*) indoor and outdoor environments, *b*) socioeconomic and parental risk factors, and *c*) nutrition were derived from self-reported questionnaires or from inspections by trained technicians (for timetable of assessments, see Supplemental Material, Table S3). Using standardized protocols, technicians carried out a home assessment when infants were 3–4 months old and gathered information about residential characteristics and activity patterns of the occupants. Secondhand smoke (SHS) exposure and presence of leaks, mold, pets, and insects and other pests were assessed by questionnaires at four time points (at time of enrollment and at 3, 6, and 12 months of age).

Three measures of socioeconomic status (SES) were examined using the father’s and mother’s education status and the household income reported at enrollment.

Information on maternal history of asthma, smoking (before/during pregnancy), SHS exposure during pregnancy, and prior live births was obtained at enrollment by questionnaire. Additionally, birth chart data provided information on type of delivery (vaginal vs. cesarean section), length of gestation, and child’s sex.

Information on breastfeeding/feeding practices was provided by the mother in postnatal questionnaires (approximately at 3, 6, and 12 months). Breastfed participants were defined as having been breastfed when the mother answered positively at any time point. Children with formula, soy, and cow’s milk intake were similarly derived. Variables relating to the introduction of solid foods were derived using any positive answer to the same question across all three questionnaires asking mothers to “indicate which foods you currently feed your child.” Timing of food introduction was not considered in this investigation.

*Statistical analysis*. Before investigating the association between TRAP and atopy, we assessed bivariate associations between sensitization at 1 year and all covariates mentioned above from the environmental (furry pets, environmental tobacco smoke, leaks, mold, pests), parental (parity, maternal asthma, maternal atopic status, maternal smoking status during pregnancy, type of delivery), SES (income, maternal and paternal education), and diet risk factors (breastfeeding, formula, soy milk, and introduction of different solid foods) for each of the three outcomes of interest (sensitization to any allergens, to any inhalant allergens, and to any food allergens), as well as sex and presence of an attached garage, to test for potential confounding. In these analyses, we first examined covariates for each time point separately. Subsequently, never/ever variables were generated whereby for any positive response to a risk factor, the participant was categorized as having this risk factor. Further bivariate analyses, evaluating the associations with each outcome separately (sensitization to any allergens, to any inhalant allergen, and to any food allergen), were carried out with the latter never/ever variables. Covariates that were significant predictors (*p* < 0.05) of each of the three outcomes of interest from the bivariate analyses were considered in models of NO_2_ and atopy for each time window. We performed a manual stepwise backward multiple variable regression with city as random intercept to obtain a parsimonious model with significantly (*p* < 0.05) associated predictors of each outcome.

In the pregnancy time period, the model for inhalant allergens included presence of an attached garage and mold, whereas the food allergens model included maternal atopy, presence of furry pets, and household income. In the first year of life, inhalant models controlled for presence of furry pets and any consumption of nuts, whereas the food allergen model controlled for maternal atopy, presence of furry pets, and any consumption of eggs, processed cereals, and peanuts. For sensitization to any allergen, the significant predictors were similar to those obtained for the food allergen model in the first year analysis. During pregnancy, the model for this outcome included maternal atopy and presence of furry pets.

The main analysis, which included a random study center intercept to adjust for the clustering within city, focused on predicted outdoor NO_2_ exposure at the home address(es) only, while we assessed time spent away from home, daycare attendance, and infiltration in sensitivity analyses. Additional analyses for effect modification by parity, sex, and maternal atopy were carried out. Predictors for these analyses were the same as those used in the main analysis. Effect estimates are presented for a 10-μg/m^3^ increase in NO_2_, approximately representing the overall standard deviation.

## Results

*Population characteristics and atopy*. Atopy data were examined for 2,482 children who had been assessed at age 1 year by 15 October 2013. Five children were excluded from the analysis due to noninterpretable skin-prick test results (*n* = 2), antihistamine medication taken before skin testing (*n* = 1), or test postponement (*n* = 2). Of the 2,477 participants, 400 infants were sensitized to at least one of the 10 tested allergens (Table 1).

**Table 1:** Atopic outcomes (inhalant, food, and any allergies) by city among 2,477 CHILD participants with valid skin-prick test at age 1 year [*n* (%)].

City (*n*, percent sensitized)	Inhalant sensitization	Food sensitization	Any sensitization
Vancouver (575, 23%)	55 (42)	92 (30)	132 (33)
Edmonton (641, 17%)	28 (21)	85 (28)	108 (27)
Winnipeg (680, 9%)	13 (10)	50 (16)	60 (15)
Toronto (581, 17%)	36 (27)	82 (27)	100 (25)
Total (2,477, 16%)	132 (5)	309 (12)	400 (16)
Data are shown as *n*, number of sensitized children (%, proportion within each atopy outcome examined).			

Only participants with nonmissing exposure and health data (pregnancy: *n* = 2,123; first year: *n* = 2,173) as well as completed questionnaire information about environmental, parental, socioeconomic, and nutritional risk factors were considered for analysis. (For a list of covariates, see Supplemental Material, Table S4.) Pregnancy and first year of life subsets used for analysis and the full cohort with sensitization results (*n* = 2,477) were virtually identical in the distribution of predictors and disease prevalence (data not shown). We also examined the effect of the exposure imputation, which was performed to include participants residing outside of the LUR surfaces. Rates of allergic sensitization were not statistically different between children assigned LUR estimates (16%) and those who had imputed concentrations (13.5%). Covariates considered for the analysis between TRAP and atopy risk were similarly distributed in the sample with imputed concentrations and that with LUR-derived exposure estimates (data not shown).

Most mothers were highly educated (93% completed at least some university or college education or more), nonsmokers during pregnancy (97%), and nonasthmatics (77%), yet with a high prevalence of sensitization to at least 1 of the 10 allergens tested (61%), and had a vaginal delivery (65%). About half of mothers had previous live births (47%). The majority of participating families (70%) belonged to higher SES based on household income ≥ $80,000/year (Table 2).

**Table 2:** Cohort characteristics among 2,477 children at 1 year of age with valid skin allergy tests, and crude odds ratio (OR) for sensitization to any allergens with 95% confidence interval (95% CI).

Characteristic	*n* (%)	Non-atopic	Atopic	OR (95% CI)
Personal/maternal covariates
Sex
Male	1,282 (52)	1,067 (51)	215 (54)	0.94 (0.76, 1.18)
Female	1,195 (48)	1,010 (49)	185 (46)
Maternal atopy status^*a*^
Yes	1,509 (61)	1,227 (59)	282 (71)	1.68 (1.33, 2.12)
No	966 (39)	848 (41)	118 (30)
Missing	2	0	2
Maternal asthma status
Yes	522 (23)	435 (23)	87 (23)	1.09 (0.83, 1.42)
No	1,771 (77)	1,485 (77)	286 (77)
Missing	184	157	27
Maternal smoking in pregnancy
Yes	74 (3)	66 (3)	8 (2)	0.74 (0.35, 1.57)
No	2,219 (97)	1,854 (97)	365 (98)
Missing	184	157	27
Maternal smoking ≥ 1 year
Yes	617 (27)	527 (27)	90 (24)	0.85 (0.65, 1.10)
No	1,674 (73)	1,391 (73)	283 (76)
Missing	186	159	27
Parity
Has previous births	1,075 (47)	914 (48)	161 (43)	0.85 (0.68, 1.07)
No previous births	1,219 (53)	1,007 (52)	212 (57)
Missing	183	156	27
Delivery mode
Vaginal	1,481 (65)	1,244 (75)	237 (73)	1.05 (0.79, 1.37)
Cesarean section	501 (22)	413 (25)	88 (27)
Missing	495	420	75
Socioeconomic covariates
Maternal education
High school	166 (7)	144 (7)	22 (6)	Reference
College or university	1,686 (74)	1,415 (74)	271 (73)	0.99 (0.61, 1.59)
Postgraduate education	439 (19)	362 (19)	77 (21)	0.98 (0.58, 1.67)
Missing	186	156	30
Household income (Can$)
< 40,000	155 (7)	132 (8)	23 (7)	Reference
40,000–80,000	490 (24)	417 (24)	73 (22)	0.96 (0.58, 1.61)
80,000–150,000	928 (45)	777 (44)	151 (46)	0.95 (0.59, 1.55)
> 150,000	511 (25)	431 (25)	80 (24)	0.83 (0.49, 1.40)
Missing	393	320	73
Environmental^*b*^ covariates
Furry pets
Yes	1,134 (65)	977 (54)	157 (44)	0.72 (0.58, 0.90)
No	1,048 (22)	846 (46)	202 (56)
Missing	295	254	41
Garage
Yes	751 (65)	623 (41)	128 (45)	1.35 (1.03, 1.79)
No	1,070 (22)	912 (59)	158 (55)
Missing	656	542	114
Introduced food in first year^*c*^
Dairy products
*Yes*	1,944 (95)	1,639 (96)	305 (92)	0.51 (0.32, 0.79)
*No*	102 (5)	75 (4)	27 (8)
*Missing*	431	363	68
Processed cereals
Yes	1,661 (83)	1,413 (85)	248 (76)	0.58 (0.44, 0.76)
No	334 (17)	256 (15)	78 (24)
Missing	482	408	74
Eggs
Yes	1,742 (85)	1,478 (86)	264 (80)	0.54 (0.40, 0.73)
No	303 (15)	235 (14)	68 (20)
Missing	432	364	68
Nuts
Yes	625 (31)	1,180 (69)	240 (72)	0.72 (0.55, 0.93)
No	1,420 (69)	533 (31)	92 (28)
Missing	432	364	68
Peanuts
Yes	941 (46)	816 (48)	125 (38)	0.63 (0.49, 0.79)
No	1,102 (54)	896 (52)	206 (62)
Missing	432	365	69
Data are shown as *n* (%). The percentages are calculated using the number of observations with known values as the denominator. ^***a***^Positive skin-prick test response to any of the allergens tested. ^***b***^Environmental covariates are based on any self-reported positive response during pregnancy and at 3, 6, or 12 months. ^***c***^Information on feeding practices are based on any self-reported positive response at 3, 6, or 12 months.				

Four hundred of 2,477 (16%) infants with a valid skin-prick test were atopic by the age of 1 year to at least one of the administered allergens. Across all cities, 309 (12.5%) infants were sensitized to any food allergen, while 132 (5.3%) had a positive response to any inhalant allergens. Vancouver had the largest proportion of atopic children (23.5%), followed by Toronto and Edmonton (both 17%), and only 9% in Winnipeg (Table 1). As expected for infants, prevalence of individual allergens was low (see Supplemental Material, Table S5), precluding the investigation of associations between NO_2_ and individual allergens. However, in initial bivariate analysis, dog [odds ratio (OR) = 1.20; 95% confidence interval (CI): 1.01, 1.21], *Der p* (OR = 1.14; 95% CI: 1.07, 1.27), and peanut sensitization (OR = 1.07; 95% CI: 1.03, 1.11) were individually associated with temporally adjusted exposure at the birth address.

Compared with atopic participants, nonatopic children were more likely in their first year to consume dairy products, eggs, nuts, peanuts, grains, and processed cereals and to reside in a home with pets and less likely to have a garage attached to their home (Table 2). Children of atopic mothers were more likely to be atopic than children of nonatopic mothers (crude OR = 1.68; 95% CI: 1.33, 2.12).

*Exposure levels and association with atopy development*. Exposure estimates were unavailable for 12% of the 2,477 children with skin-prick test data, because 173 participants had homes located > 50 km from the city center, and 131 did not provide their residential history since enrollment.

Among the 2,173 participants with complete residential histories who resided within the 50-km buffer, 252 homes fell outside of each city core limits and were assigned imputed NO_2_ concentrations. Mean exposure levels differed significantly by city, ranging from 28 μg/m^3^ in Toronto to 9.9 μg/m^3^ in Winnipeg (Table 3). After applying the bi-weekly temporal adjustment and accounting for residential mobility (83% did not change their address), estimated exposures were lower across all cities due to decreasing ambient concentrations between the development of the LUR model and the date of birth (Brook et al. 2014), with a greater decline for older LUR surfaces. The difference between these estimates is not likely attributable to address changes, but mostly to the temporal adjustment from original LUR models to the time of the present investigation. When examining the pairwise differences of spatiotemporal bi-weekly means by time window, we found significant differences (*p* < 0.05) between exposure during pregnancy and the first year of life, unlike the nonsignificant differences obtained with estimates not accounting for residential mobility (*p* = 0.3).

**Table 3:** NO_2_ exposure (μg/m^3^) levels of participants with complete health information, unadjusted and adjusted for temporal trend and residential mobility, in each time window by city.

Variable	Pregnancy		First year	
	Based on address at enrollment^*a*^	Temporally adjusted using all addresses	Based on address at birth^*a*^	Temporally adjusted using all addresses
Edmonton (*n* = 554)
Mean ± SD	26.3 ± 8.5	24.1 ± 8.8	26.1 ± 8.6	24.0 ± 8.8
Median	27.3	24.4	27.2	24.8
Range	10.3–45.8	6.9–50.7	10.3–50.2	7.6–49.3
Toronto (*n* = 496)
Mean ± SD	37.2 ± 9.3	28.1 ± 7.9	36.9 ± 9.3	28.2 ± 7.7
Median	36.1	26.7	35.3	25.2
Range	17.7–78.8	12.7– 60.9	17.6–78.6	12.0–59.4
Vancouver (*n* = 543)
Mean ± SD	36.2 ± 8.3	23.6 ± 6.4	35.9 ± 8.4	23.8 ± 6.1
Median	35.2	22.5	35.1	29.5
Range	11.8–58.9	7.2–47.3	11.8–58.8	7.3–47.2
Winnipeg (*n* = 580)
Mean ± SD	16.5 ± 5.7	9.4 ± 4.0	16.4 ± 5.7	9.9 ± 3.6
Median	16	9	15.9	7.5
Range	3.9–30.3	1.2–29	2.3–28.9	1.1–17.3
*n*, number of participants with at least 75% exposure coverage. ^***a***^LUR estimates with no adjustment for temporal variation.				

**Figure 1:**
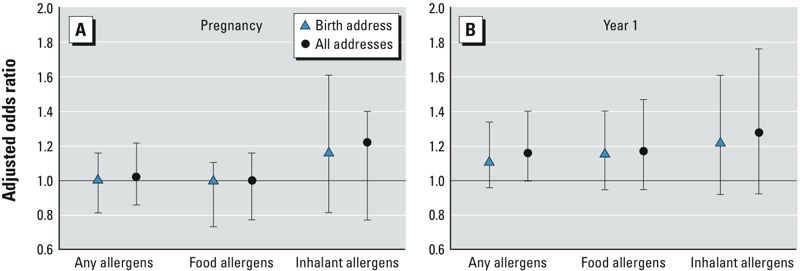
Adjusted odds ratio for risk of atopy per 10-μg/m^3^ increase in NO_2_ exposures temporally adjusted at birth address and temporally adjusted and accounting for residential mobility. (*A*) During pregnancy: inhalant allergens model controlled for presence of an attached garage and mold (*n *= 1,836); food allergens model controlled for mother’s atopic status, presence of furry pets, household income (*n *= 1,913); any allergens model (*n *= 2,123) controlled for mother’s atopic status, and presence of furry pets. (*B*) During the first year of life: inhalant model (*n *= 2,058) controlled for presence of furry pets and any consumption of nuts since birth; food allergen analysis (*n *= 2,002) adjusted for mother’s atopic status, presence of furry pets, and any consumption of eggs, processed cereals, and peanuts; any allergen analysis (*n *= 2,173) adjusted for mother’s atopic status, presence of furry pets, consumption of eggs, processed cereals, and peanuts.

Compared with TRAP exposure estimated at the birth address with no temporal adjustment, NO_2_ estimates that incorporated temporal variability in ambient concentrations increased the magnitude of the effect estimates for the first year of life analysis [any allergens: adjusted odds ratio (aOR) = 1.10; 95% CI: 0.96, 1.34], yet without reaching statistical significance (see Supplemental Material, Table S6). Further, estimates of effect generally increased when temporally adjusted models further accounted for mobility (Figure 1). However, the increased spatial resolution also led to larger confidence intervals around the atopy risk estimates. During the first year of life, NO_2_ was associated with sensitization to any allergen tested at 1 year of age (aOR = 1.16; 95% CI: 1.00, 1.41) when considering temporally adjusted exposures that also accounted for residential mobility. When examining each group of allergens separately, we also found positive, but nonsignificant, associations (aOR = 1.17; 95% CI: 0.95, 1.47 for inhalant allergies and aOR = 1.27; 95% CI: 0.93, 1.76 for food allergies) (Figure 1B). In contrast, during pregnancy (Figure 1A), effect estimates were null for sensitization to any allergens (aOR = 1.02; 95% CI: 0.86, 1.22) and for sensitization to food allergens (aOR = 1.00; 95% CI: 0.77, 1.61). In this time window, the association between exposure and inhalant allergens atopy was nonsignificant (aOR = 1.18; 95% CI: 0.77, 1.61).

**Figure 2:**
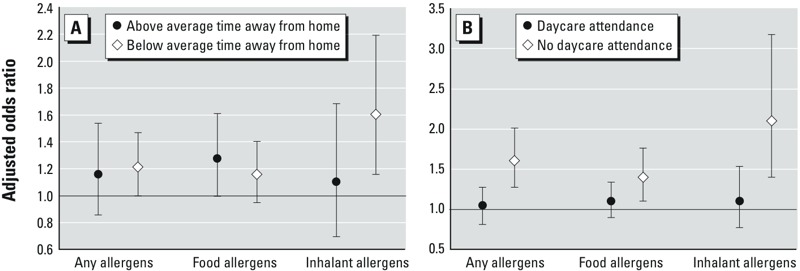
Adjusted odds ratio of atopy per 10-μg/m^3^ NO_2_ increase during the first year stratified by (*A*) time–activity patterns (defined by the city-specific median hours per day based on the three questionnaires submitted after birth around 3, 6, and 12 months) among children spending more time (*n *= 976) and those spending less time (*n *= 1,026) away from the home; and (*B*) daycare facilities attendance among daycare attendees (*n *= 765) and children never attending daycare (*n *= 1,236). Models are adjusted for the same covariates as in the main analysis (Figure 1B).

Analyses of the effect of greater or lesser time spent away from the home indicated improved precision in estimates among children spending more time at the home, and identified a potential source of exposure misclassification (Figure 2A). Participants (*n* = 976) who spent more time away from the home (median, 3.3 hr/day for all cities) generally had slightly smaller effect estimates with larger confidence intervals (any allergens: aOR = 1.16; 95% CI: 0.85, 1.53) than those spending less time (*n* = 1,026) away from their home addresses (aOR = 1.22; 95% CI: 1.00, 1.47). This association was likely driven by the sensitization to inhalant allergens (children spending the city-specific median time or less away from home: aOR = 1.61; 95% CI: 1.15, 2.19 vs. those spending more than the city-specific median time away from their homes: aOR = 1.10; 95% CI: 0.69, 1.68).

Stratifying the cohort by daycare attendance (35% attended daycare) also suggested a source of exposure misclassification, as non-daycare attendees (*n* = 1,236) had 61% increased odds of developing atopy (aOR = 1.61; 95% CI: 1.28, 2.01), whereas the risk was smaller and not significant in children who did attend daycare (*n* = 765; aOR = 1.05; 95% CI: 0.81, 1.28) (Figure 2B). The sensitization to inhalant allergens showed the largest association with NO_2_ exposure (aOR = 2.1; 95% CI: 1.40, 3.17 for children never attending daycare vs. aOR = 1.10; 95% CI: 0.77, 1.54 for daycare attendees). Given these results, we investigated whether exposures other than NO_2_ (e.g., contact with other children) may play a role in the sensitization onset. Thus, we ran a stratified analysis by presence of siblings that showed that participants who were in households with other siblings had lower odds of developing sensitization to any allergens (*n* = 874; aOR = 1.16; 95% CI: 0.91, 1.54) following exposure to TRAP than those with no siblings (*n* = 1,085, aOR = 1.28; 95% CI: 1.0, 1.54) (see Supplemental Material, Figure S1).

For a 10-μg/m^3^ increase in NO_2_ exposure, the odds of sensitization to any allergen for children living in homes with greater ventilation (*n* = 687) was slightly higher (aOR = 1.22; 95% CI: 0.91, 1.61) than for children living in tighter homes (*n* = 824, aOR = 1.10; 95% CI: 0.82, 1.47) during the heating season, but not in the warm season (see Supplemental Material, Figure S2).

## Discussion

In this prospective multi-center birth cohort study, exposure to NO_2_ during the first year of life, but not during pregnancy, was positively associated with atopy at age 1 year. To our knowledge, this is the first birth cohort study where atopy in relation to traffic-related air pollution was determined in the first year of life (Brauer et al. 2007; Gruzieva et al. 2012; Nordling et al. 2008). Positive associations between NO_2_ and specific sensitization to common food, but not inhalant allergens, were observed in a subgroup of 700 Dutch children from the PIAMA (Prevention and Incidence of Asthma and Mite Allergy) cohort at age 4 (Brauer et al. 2007). In the Swedish BAMSE (Children, Allergy, Milieu, Stockholm, Epidemiology) cohort, exposure during the first year of life was associated with an increased risk of only pollen sensitization at age 4 years (no association with food allergens) (Gruzieva et al. 2012).

The ability to refine individual estimates of exposure to TRAP by incorporating temporal changes in air pollution concentrations and in participants’ residential mobility led to larger effect estimates; however, the improvement in precision of these effects was negligible and not consistently improved across all three outcomes. The exposure assessment was derived from modeled estimates rather than measurements, increasing error propagation in the estimates used for evaluating the association with atopy outcomes. However, the correlation between the exposures during the entire pregnancy and the first year of life decreased with more specific exposure measures, suggesting that refined exposure assessment enables improved differentiation between exposure periods. This finding is supported by a study showing that temporally updated (based on air dispersion model data) LUR models provide accurate exposure estimates (Mölter et al. 2010).

In light of the recently published European meta-analysis of air pollution with allergic sensitization (Gruzieva et al. 2014), we explored potential effect modification by sex or maternal atopy. Similar to the results of this meta-analysis, we found no effect modification by sex. However maternal atopy showed borderline significant effects for exposure during pregnancy and smaller magnitude of effect compared with the main model in the first year (aOR for maternal atopy × NO_2_ interaction term = 1.04; 95% CI: 0.98, 1.10 during pregnancy and aOR = 1.04; 95% CI: 1.00, 1.10 during the first year).

We demonstrated stronger associations between TRAP and atopy in our stratified analyses when daycare attendance and individual time–activity patterns were considered. In particular for exposures during the first year of life, when inhalant allergen sensitization was considered separately, participants for whom exposure misclassification was less likely (i.e., those spending more than the city median time at home, and those who did not attend daycare) had stronger associations. In a small subsample of participants providing daycare addresses (*n* = 235), exposures were not significantly different in homes and in daycares (data not shown), making it unlikely that lower exposures outside the homes would explain reduced effects among those attending daycare. Although the observed differences in these subanalyses could also be attributable to more variability suggestive of a classical exposure error, we explored the possibility that the differences observed were related to exposure other than TRAP. Odds ratios of sensitization to any allergens were lower for participants spending more time in the home or not attending daycare compared with daycare attendees or those spending more time away from the home for the same rate of TRAP increase, suggesting that this latter group might be exposed to other exogenous protective exposure, such as presence of other children. The additional stratified analysis by presence of siblings seemed to support the argument that exposure to other children is likely to play a protective role in the development of atopy.

Along with refined exposure assessment modeling, major strengths of our study are the prospective design from early in pregnancy and the objective definition of sensitization. Comparisons between the few birth cohorts examining perinatal exposures to traffic pollution are complicated by the various definitions of atopy or allergic sensitization, most often assessed by self-reported symptoms (Bråbäck and Forsberg 2009), which can lead to misclassification of outcomes. In the present study, atopic status was based on objective skin-prick tests using a common protocol for all participants. Gathering questionnaire and home inspection data enabled us to collect extensive individual data on known and suspected risk factors about indoor and outdoor environments, and parental health status, as well as detailed dietary information that are seldom acquired in large cohort studies as early as in this investigation. However, the number of missing covariates is a limitation as sample sizes for individual analyses were substantially reduced.

Despite the advantage of multiple questionnaires and detailed home assessments, the use of self-reported information on environmental risk factors, which may be biased by parental health status, is a concern. Although the cohort was unselected and the prevalence of parental allergy and current asthma similar to that in the Canadian population, there is a bias toward higher SES compared with the general population, as is often the case with birth cohorts. Further, although this is one of few analyses of TRAP to examine the role of infiltration, our assessment was limited by the use of a model for particle infiltration developed for cities in the United States (Allen et al. 2012) to classify infiltration of TRAP in Canadian homes. We mapped variables in the MESA Air cohort questionnaires to the most similar questions available in CHILD; however, it is likely that we introduced some error in recoding the CHILD variables and thus in developing infiltration estimates, which are already difficult to model based on actual measurements. In addition, the model was developed in U.S. cities spanning a wider north–south geographical area, and consequently developed for a hotter climate, which led to a temperature threshold variable (23°C) that might differ from the cut-off obtained using Canadian data. In the case of the cold season infiltration models, we found the expected differences between homes with participants in the “leaky” homes showing stronger associations between TRAP exposure and sensitization to any allergens only. Lack of sufficient power precluded the identification of differences in this analysis. Future studies should consider an infiltration measurement substudy to develop a study-specific model.

Children at 1 year of age developed more sensitization to food (12.5%) than inhalant allergens (5.5%), similar to findings in the European birth cohorts in which participants were older and showed higher prevalence rates of sensitization [BAMSE with 16% and 15% (Gruzieva et al 2012) and PIAMA with 23.9% and 8.5% (Gehring et al. 2010) for food and inhalant allergens, respectively]. However, we observed that exposures during first year of life may contribute differently to the potential load of sensitization. In conclusion, this study demonstrates that in cities with low levels of ambient traffic-related air pollution, incorporating different tools (GIS, monitoring data, questionnaires, and home environmental assessment) to account for temporal variation, residential history, and time–location patterns in the estimation of individual-level exposures can help clarify the association between perinatal exposure to traffic-related air pollution and the development of allergic sensitization to common inhalant and food allergens.

## Supplemental Material

(551 KB) PDF
