# Supplementary material for: Perinatal Exposure to Traffic-Related Air Pollution and Atopy at 1 Year of Age in a Multi-Center Canadian Birth Cohort Study
Source: Environ Health Perspect. 2015 Mar 31;123(9):902–8. doi: 10.1289/ehp.1408700 (PMC4559953; doi:10.1289/ehp.1408700)
Supplement: (551 KB) PDF [file ehp.1408700.s001.acco.pdf]

Environ Health Perspect
doi: 10.1289/ehp.1408700

Note to Readers: ***EHP*** **strives to ensure that all journal content is accessible to all readers. However, some figures and Supplemental Material published in *EHP* articles may not conform to 508 standards due to the complexity of the information being presented. If you need assistance accessing journal content, please contact ehp508@niehs.nih.gov. Our staff will work with you to assess and meet your accessibility needs within 3 working days.**

Supplemental Material

Perinatal Exposure to Traffic-Related Air Pollution and Atopy at 1 Year of Age in a Multi-Center Canadian Birth Cohort Study

Hind Sbihi, Ryan W. Allen, Allan Becker, Jeffrey R. Brook, Piush Mandhane, James A. Scott, Malcolm R. Sears, Padmaja Subbarao, Tim K. Takaro, Stuart E. Turvey, and Michael Brauer

Table of Contents

Table S1: LUR models for nitrogen dioxide (variables[a], estimated annual means for all participants and standard deviation (sd)).

Table S2: Particle Infiltration (Finf) variables; mapping questions used in the MESA-Air (Allen et al. 2012) and CHILD cohorts.

Table S3: Timetable of assessments in the Canadian Healthy Infant Longitudinal Development (CHILD) study used in the investigation of atopy in relation to traffic-related air pollution.

Table S4: Crude Odds Ratios (OR) and 95% Confidence Interval (95% CI) of sensitization to any, inhalant and food allergens in relation to participants' physical environment characteristics, maternal factors, and nutrition at different time points.

Table S5: Proportion of positive responses to individual allergen skin prick tests by CHILD city (in (%)).

Table S6: Adjusted Odds Ratios (aOR) for risk of atopy per 10 μg/m^3 increase in NO_2 exposures during pregnancy and the first year of life.

Figure S1: Adjusted OR of risk of atopy in CHILD families for a 10 μg/m^3 increase in NO_2 during the first year of life, stratified by presence of siblings in participants families (group with

no siblings, n= 1085; group with sibling, n= 874). All models are adjusted for the same covariates as those used in the main analysis (Figure 1B).

Figure S2 - Adjusted Odds Ratio of risk of atopy for 10 μg/m^3 increase in NO2 during the first year of life stratified by season (defined using weekly average of 18°C as cutoff to define cold and warm) and by home PM infiltration status (defined based on city-specific 80th percentile predicted household PM infiltration efficiency; "leaky" homes: n= 687; "sealed" homes: n=824). All models are adjusted for the same covariates as in the main analysis (Figure 1B).

References

Table S1: LUR models for nitrogen dioxide (variables[a], estimated annual means for all participants and standard deviation (sd)).

City	Land Use Regression Model	R^2	Mean[b] (sd) ($\mu g/m^3$)
Edmonton (Allen et al. 2011)	NO_2 = 16.60 + 0.02(IND_1500) + 0.30(RD1_1000) + 12.01(RD2_50) + 4.66(RD.100) + 0.06(WTR.1000) + 0.58(Distance City Center)	0.81	22.2 (9.1)
Winnipeg (Allen et al. 2011)	NO_2= 4.11 + 0.007(IND.2000) + 0.33(IND_200)+ 7.14(HM_50) + 5.64(RD.75)+ 0.09(POP_2500) + 0.18(Y)	0.77	8.6 (5.0)
Vancouver (Henderson et al. 2007)	NO_2= 42.6 + 10.5(RD1_100) + 0.274(RD1_1000) + 4.24(RD2_200) + 0.074(POP_2500) + 0.116(COM) – 0.02(ELEV) – 0.591(X)	0.76	21.2 (8.9)
Toronto (Jerrett et al. 2007)	Log(NO_2) = 8 + 0.18(RD1_200) + 0.6(RD2_50) + 0.0016(IND.750) + $8.3*10^{-5}$(DC2000)- $8*10^{-6}$(X) + 0.13(D_WIND.1500) + 0.001(TRAF.500)	0.67	27.1 (8.9)

[a]All the buffer zones radii for the derived land use variables are indicated in suffix. IND: industrial; WTR: Water; COM: commercial; RD1: Highway; RD2: Major Road; RD: all roads; HM: Highway or Major Road; ELEV: Elevation; POP: population density; DC: density of dwellings; TRAF: Traffic counts; D_WIND: downwind of major expressways; Y: Latitude; X: longitude. [b]The temporally adjusted annual concentrations and standard errors in $\mu g/m^3$ are presented for each city.

Table S2: Particle Infiltration (Finf) variables; mapping questions used in the MESA-Air (Allen et al. 2012) and CHILD cohorts.

Season	MESA-Air Variable	CHILD Variable	Partial R^2 in the MESA-Air model
Warm (≥18°C)	Central AC used > ½ time in past July	Central AC used regularly in the summer[a]	0.560
	Windows open ≥ ½ time in past summer	Windows open ≥ 1hr more than 2 times/week in mid-summer[a]	0.080
	Central AC used > ½ time in past July and 2-week avg. outdoor temperature > 23°C	Central AC used regularly in the summer [c] and 2-week government monitors average[b]	0.051
	Central AC used a few days in past July	Central AC use occasionally in the summer[a]	0.013
	2-week avg. outdoor temperature > 23°C	2-week avg. outdoor temperature > 23°C[b]	0.000
Cold (<18°C)	2-week avg. outdoor temperature (°C)	2-week avg. outdoor temperature[b]	0.222
	Home has forced air heat	Home has furnace[c]	0.166
	Windows open ≥ ½ time in past summer	Windows open ≥ 1hr more than 2 times/week in mid-summer[a]	0.069

[a]Home environmental questionnaire. [b]Environment Canada (http://climate.weather.gc.ca/). [c]Home inspection.

The table above displays how questionnaire and home inspection variables were selected to replicate the MESA-Air model in order to predict a house- and season-specific infiltration. These predictions were used to derive for each season (where hot and cold season were defined using 18°C cut-off,) different percentiles of particle infiltration by city. The 80th percentile was chosen as cut-off to stratify homes with greater ventilation (“leaky”) from tighter homes (“sealed”).

Table S3: Timetable of assessments in the Canadian Healthy Infant Longitudinal Development (CHILD) study used in the investigation of atopy in relation to traffic-related air pollution.

Time point	Data collection
Pregnancy<br>(18+ weeks)	Maternal, paternal demographics;<br>Maternal stress questionnaires ;<br>Detailed environmental questionnaires.
Pregnancy<br>(~36 weeks)	Maternal stress questionnaires repeated.
Birth	Delivery outcomes
~3months	Home visit: home assessment;<br>Detailed environmental questionnaires<br>Child health and nutrition questionnaires,
~6 months	Web-based or mail-out questionnaires: environmental update, child health and nutrition
~1 year	Maternal and child allergy skin tests;<br>Detailed environmental questionnaire;<br>Child health and nutrition questionnaires,

Table S4: Crude Odds Ratios (OR) and 95% Confidence Interval (95% CI) of sensitization to any, inhalant and food allergens in relation to participants' physical environment characteristics, maternal factors, and nutrition at different time points.

		Any allergens		Inhalant allergens		Food allergens	
**Covariates**	**N[a]**	**OR**	**(95% CI)**	**OR**	**(95% CI)**	**OR**	**(95% CI)**
**Parental**							
Mother atopy	2475	1.68	(1.33, 2.12)	1.45	(1.0, 2.12)	1.70	(1.31, 2.21)
Mother education[b]	2084	1.10	(0.95, 1.09)	0.93	(0.82, 1.05)	1.11	(0.91, 1.37)
Father education[b]	2271	1.06	(0.95, 1.10)	1.03	(0.93, 1.16)	1.01	(0.82, 1.18)
Household income[c]	2084	0.97	(0.84, 1.11)	1.06	(0.84, 1.35)	0.93	(0.80, 1.09)
Mother self-reported asthma	2293	1.09	(0.84, 1.41)	0.93	(0.59, 1.45)	1.10	(0.83, 1.46)
Mother smoking pregnancy	2293	0.74	(0.35, 1.57)	1.65	(0.70, 3.92)	0.67	(0.33, 1.36)
Mother smoking (≥ 1 year)	2291	0.85	(0.65, 1.10)	0.93	(0.61, 1.41)	0.84	(0.16, 1.24)
Other siblings	2294	0.85	(0.67, 1.07)	0.82	(0.56, 1.17)	0.81	(0.61, 1.07)
Delivery[d]	2130	1.02	(0.81, 1.25)	1.02	(0.96, 1.09)	0.92	(0.72, 1.19)
**In Utero, Environment**							
Furry Pets	2126	0.71	(0.56, 0.90)	0.93	(0.55, 1.57)	0.72	(0.48, 0.90)
Leaks	2122	0.95	(0.73, 1.23)	0.74	(0.47, 1.17)	0.88	(0.68, 1.16)
Second hand smoke	2291	1.01	(0.72, 1.42)	0.69	(0.40, 1.17)	1.03	(0.70, 1.51)
**Postnatal, Environment**							
Furry pets at 3 months	2258	0.89	(0.65, 1.24)	1.53	(1.04, 2.27)	0.69	(0.53, 1.01)
Furry pets at 6 months	1956	0.78	(0.43, 1.02)	1.42	(0.91, 2.21)	0.78	(0.60, 1.02)
Furry pets at 1 year	2041	0.79	(0.56, 1.11)	1.4	(0.94, 2.07)	0.89	(0.68, 1.16)
Furry pets (anytime point)	2182	0.72	(0.58, 0.90)	1.3	(0.90, 1.88)	0.63	(0.49, 0.80)
Pests and bugs at 3months	2306	1.02	(0.81, 1.28)	0.75	(0.44, 1.26)	1.04	(0.80, 1.35)
Pests and bugs at 1year	2043	0.89	(0.67, 1.19)	0.71	(0.37, 1.36)	0.88	(0.63, 1.21)
Pests and bugs(anytime point)	2341	0.98	(0.78, 1.23)	0.72	(0.44, 1.20)	0.95	(0.74, 1.23)
Mold at 3 months	2280	1.07	(0.85, 1.35)	0.5	(0.29, 0.88)	1.10	(0.85, 1.43)
Mold at 1year	814	0.56	(0.17, 1.86)	0.67	(0.29, 1.53)	0.70	(0.33, 1.49)
Mold (any time point)	2311	1.08	(0.86, 1.36)	1.12	(0.76, 1.62)	1.05	(0.81, 1.35)
Leaks at 3 months	2280	1.03	(0.79, 1.33)	1.24	(0.82, 1.89)	0.98	(0.73, 1.33)
Leaks at 1 year	2043	0.82	(0.58, 1.14)	1.18	(0.70, 1.95)	0.69	(0.46, 1.04)
Leaks (any time point)	2128	0.87	(0.69, 1.10)	0.94	(0.64, 1.38)	0.83	(0.64, 1.07)
Attached garage at 3 months	1855	1.08	(0.83, 1.39)	0.77	(0.49, 1.19)	1.24	0.93 - 1.65
Attached garage at 1year	1659	1.30	(1.00, 1.71)	1.30	(0.83, 2.03)	1.29	0.96 - 1.74
Attached Garage	1821	1.35	(1.03, 1.79)	1.55	(1.00, 2.50)	1.41	(1.04, 1.91)
**Nutrition**							
Breastfeeding at birth	2263	1.63	(0.78, 3.42)	1.92	(0.46, 7.95)	1.26	(0.46, 7.95)
Formula at birth	2263	0.84	(0.66, 1.07)	0.97	(0.66, 1.43)	0.76	(0.52, 1.09)
Breastfeeding ever	2345	1.14	(0.67, 1.97)	1.22	(0.44, 3.40)	0.87	(0.51, 3.95)
Formula ever	2345	0.84	(0.68, 1.04)	0.83	(0.58, 1.20)	0.87	(0.58, 1.16)
Cow milk ever	2304	1.81	(0.50, 6.61)	2.57	(0.31, 21.0)	0.65	(0.88 - 19.5)
Solid food ever	2044	0.77	(0.61, 0.99)	0.88	(0.58, 1.33)	0.78	(0.60 - 1.30)
Grains ever	2045	0.37	(0.16, 0.83)	0.64	(0.15, 2.77)	0.33	(0.14 - 0.76)
Dairies ever	2046	0.51	(0.32, 0.79)	0.98	(0.42, 2.30)	0.45	(0.28 - 0.72)
Processed cereals ever	1995	0.58	(0.44, 0.76)	0.74	(0.47, 1.16)	0.51	(0.38 - 0.69)
Eggs ever	2045	0.54	(0.40, 0.73)	0.95	(0.56, 1.62)	0.48	(0.35 - 0.66)
Shellfish ever	2043	0.8	(0.59, 1.07)	0.72	(0.45, 1.15)	0.8	(0.59 - 1.10)
Fish ever	2046	0.93	(0.71, 1.22)	1.06	(0.67, 1.62)	0.83	(0.62 - 1.11)
Meat ever	2046	0.77	(0.44, 1.35)	1.36	(0.48, 3.84)	0.63	(0.60 - 1.63)
Peanuts ever	2043	0.63	(0.49, 0.79)	0.66	(0.45, 0.97)	0.57	(0.44 - 0.75)
Nuts ever	2045	0.72	(0.55, 0.93)	0.61	(0.40, 0.93)	0.78	(0.59 - 1.04)

[a]N: indicates the total known values among the 2477 children for which skin prick test data as of October 15th, 2013 and does not include missing values. [b]Parental educational attainment was treated as an ordinal variable (1: High School, 2: College/University, 3: Post Graduate (Master, PhD, MD)). [c]Household Income was treated as an ordinal variable (1: less than $40K, 2: $40K-$80K, 3: $80K-$150K, 4: >$150K). [d]Delivery mode categories: Vaginal, C-Section, Other.

Table S5: Proportion of positive responses to individual allergen skin prick tests by CHILD city (in (%)).

City	Alt	*Der p*	*Der f*	Cat	Dog	Blatt	Peanut	Milk	Eggs	Soy
Vancouver (N = 575)	1.9	1.4	3.0	3.1	1.0	1.2	6.1	8.5	4.0	1.7
Edmonton (N = 641)	1.1	0.5	0.9	1.4	0.3	0.3	6.2	9.2	2.3	1.1
Winnipeg (N = 680)	0.1	0.1	0.1	1.2	0.1	0.4	3.2	5.3	0.4	0.1
Toronto (N = 581)	1.2	1.4	0.7	2.2	2.2	1.0	6.5	9.1	1.7	2.1

Table S6: Adjusted Odds Ratios (aOR) for risk of atopy per 10 μg/m^3 increase in NO_2 exposures during pregnancy and the first year of life.

	All Allergens		Food Allergens		Inhalant Allergens	
	aOR	95% CI	aOR	95% CI	aOR	95% CI
Pregnancy						
Exposure at birth address	1.06	0.95 – 1.28	1.05	0.87 – 1.34	1.05	0.77 – 1.40
Temporally adjusted (Birth address) exposure	1.01	0.81 – 1.16	0.99	0.73 – 1.10	1.16	0.86 – 1.61
Temporally adjusted exposure accounting for residential mobility (All addresses)	1.02	0.86 – 1.22	1.00	0.77 – 1.61	1.18	0.77 – 1.61
First Year						
Exposure at birth address	1.05	0.95 – 1.28	1.08	0.95 – 1.28	1.16	0.91 – 1.40
Temporally adjusted exposure (Birth address)	1.10	0.96 - 1.34	1.15	0.95-1.40	1.22	0.92-1.62
Temporally adjusted exposure accounting for residential mobility (All addresses)	1.16	1.00- 1.41	1.17	0.95-1.47	1.28	0.93-1.76

Model covariates:

Any allergies and NO_2 during pregnancy: mother's atopic status, presence of furry pets.

Any allergies and NO_2 during first year: mother's atopic status, presence of furry pets, consumption of eggs, consumption of processed cereals, and consumption of peanuts

Food allergies and NO_2 during pregnancy: mother's atopic status, presence of furry pets, and household income

Food allergies and NO_2 during first year: mother's atopic status, presence of furry pets, consumption of eggs, consumption of processed cereals, and consumption of peanuts

Inhalant allergies and NO_2 during pregnancy: presence of an attached garage, presence of mold.

Inhalant allergies and NO_2 during first year: presence of furry pets and consumption of nuts.

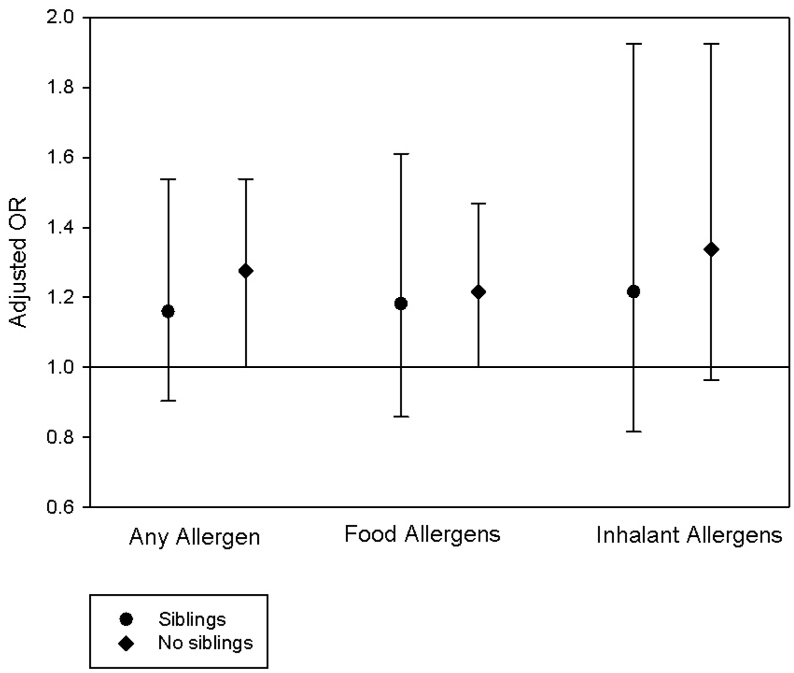

Figure S1: Adjusted OR of risk of atopy in CHILD families for a 10 μg/m^3 increase in NO_2 during the first year of life, stratified by presence of siblings in participants families (group with no siblings, n= 1085; group with sibling, n= 874). All models are adjusted for the same covariates as those used in the main analysis (Figure 1B).

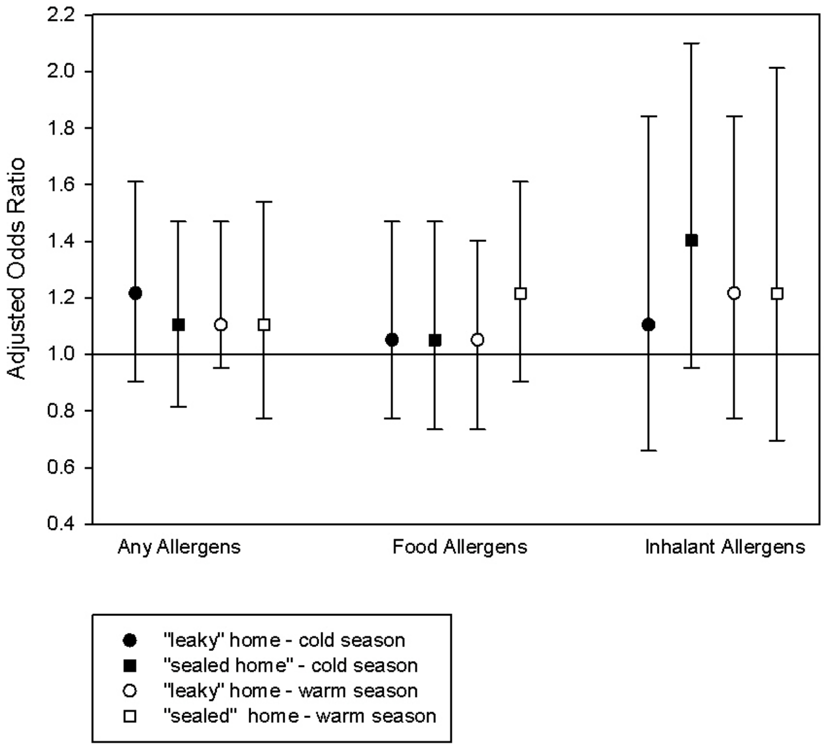

Figure S2 - Adjusted Odds Ratio of risk of atopy for 10 µg/m^3 increase in NO2 during the first year of life stratified by season (defined using weekly average of 18°C as cutoff to define cold and warm) and by home PM infiltration status (defined based on city-specific 80th percentile predicted household PM infiltration efficiency; "leaky" homes: n= 687; "sealed" homes: n=824). All models are adjusted for the same covariates as in the main analysis (Figure 1B).

References

Allen RW, Amram O, Wheeler AJ, Brauer M. 2011. The transferability of NO and NO2 land use regression models between cities and pollutants. Atmos. Environ. 45:369–378; doi:10.1016/j.atmosenv.2010.10.002.

Allen RW, Adar SD, Avol E, Cohen M, Curl CL, Larson T, et al. 2012. Modeling the Residential Infiltration of Outdoor PM2.5 in the Multi-Ethnic Study of Atherosclerosis and Air Pollution (MESA Air). Environ. Health Perspect.; doi:10.1289/ehp.1104447.

Henderson SB, Beckerman B, Jerrett M, Brauer M. 2007. Application of land use regression to estimate long-term concentrations of traffic-related nitrogen oxides and fine particulate matter. Environ. Sci. Technol. 41: 2422–2428.

Jerrett M, Arain MA, Kanaroglou P, Beckerman B, Crouse D, Gilbert NL, et al. 2007. Modeling the intraurban variability of ambient traffic pollution in Toronto, Canada. J. Toxicol. Environ. Health A 70: 200–212.
